# Investigating Citizens’ Acceptance of Contact Tracing Apps: Quantitative Study of the Role of Trust and Privacy

**DOI:** 10.2196/48700

**Published:** 2024-01-18

**Authors:** Grace Fox, Lisa van der Werff, Pierangelo Rosati, Theo Lynn

**Affiliations:** 1 Irish Institute of Digital Business Dublin City University Dublin Ireland; 2 J.E. Cairnes School of Business & Economics University of Galway Galway Ireland

**Keywords:** privacy, trust, public health surveillance, contact tracing, mobile apps, adoption, information disclosure

## Abstract

**Background:**

The COVID-19 pandemic accelerated the need to understand citizen acceptance of health surveillance technologies such as contact tracing (CT) apps. Indeed, the success of these apps required widespread public acceptance and the alleviation of concerns about privacy, surveillance, and trust.

**Objective:**

This study aims to examine the factors that foster a sense of trust and a perception of privacy in CT apps. Our study also investigates how trust and perceived privacy influence citizens’ willingness to adopt, disclose personal data, and continue to use these apps.

**Methods:**

Drawing on privacy calculus and procedural fairness theories, we developed a model of the antecedents and behavioral intentions related to trust and privacy perceptions. We used structural equation modeling to test our hypotheses on a data set collected at 2 time points (before and after the launch of a national CT app). The sample consisted of 405 Irish residents.

**Results:**

Trust in CT apps was positively influenced by propensity to trust technology (β=.074; *P*=.006), perceived need for surveillance (β=*.*119; *P*<.001), and perceptions of government motives (β=.671; *P*<.001) and negatively influenced by perceived invasion (β=−.224; *P*<.001). Perceived privacy was positively influenced by trust (β=.466; *P*<.001) and perceived control (β=.451; *P*<.001) and negatively influenced by perceived invasion (β=−.165; *P*<.001). Prelaunch intentions toward adoption were influenced by trust (β=*.*590; *P*<.001) and perceived privacy (β=*.*247; *P*<.001). Prelaunch intentions to disclose personal data to the app were also influenced by trust (β=.215; *P*<.001) and perceived privacy (β=.208; *P*<.001) as well as adoption intentions before the launch (β=.550; *P*<.001). However, postlaunch intentions to use the app were directly influenced by prelaunch intentions (β=.530; *P*<.001), but trust and perceived privacy only had an indirect influence. Finally, with regard to intentions to disclose after the launch, use intentions after the launch (β=.665; *P*<.001) and trust (β=.215; *P*<.001) had a direct influence, but perceived privacy only had an indirect influence. The proposed model explained 74.4% of variance in trust, 91% of variance in perceived privacy, 66.6% of variance in prelaunch adoption intentions, 45.9% of variance in postlaunch use intentions, and 83.9% and 79.4% of variance in willingness to disclose before the launch and after the launch, respectively.

**Conclusions:**

Positive perceptions of trust and privacy can be fostered through clear communication regarding the need and motives for CT apps, the level of control citizens maintain, and measures to limit invasive data practice. By engendering these positive beliefs before launch and reinforcing them after launch, citizens may be more likely to accept and use CT apps. These insights are important for the launch of future apps and technologies that require mass acceptance and information disclosure.

## Introduction

### Background

The outbreak of COVID-19 and the ensuing global pandemic resulted in many governments undertaking digital government transformation [1] through the introduction of public health surveillance technologies including contact tracing (CT) apps [2,3]. As a result, and unlike previous health emergencies, governments had access to an unprecedented volume, variety, and velocity of location and health data [4]. The use of such data for epidemiological surveillance can aid in decision support, accelerate case identification, interrupt community transmission, and enable public health communication [5]. Notwithstanding these benefits, the pace at which these apps have been implemented and the level of surveillance they enable have raised ethical concerns [6] and fears around privacy and public trust [7].

The success of CT apps is dependent on uptake by large populations [8], and privacy-related concerns have been positioned as a critical barrier facing government-introduced CT apps [9]. Government-introduced CT apps differ from surveillance technologies and mobile health (mHealth) apps, as they combine both location-based data and electronic personal health information (ePHI). Both these contexts, in themselves, raise significant privacy concerns, particularly with respect to potential secondary use and government intrusion [10]. Thus, the COVID-19 pandemic presents a unique empirical context to explore citizens’ perceptions of health surveillance using mobile apps that capture both location-based data and ePHI. Government-introduced CT apps constitute a new public health context. Although there is an established literature based on traditional CT, digital CT is an innovation that can only be fully explored during pandemics, and thus research opportunities are limited. Given the novel context for individuals and public health agencies, where the former engage with new or unfamiliar trust referents, it is particularly critical to explore how individuals marry competing beliefs about surveillance, trust, and government motives for introducing these technologies and how these beliefs influence their behavioral responses. Extant literature has demonstrated the importance of trust in the government in influencing CT adoption [11,12] and perceptions of CT technologies [13] and shown that privacy concerns represent a barrier to adoption [9].

### Prior Work

This study builds upon important extant research focusing on the acceptance of CT apps to delve further into the role of privacy and trust and addresses 3 gaps in the literature. First, existing studies support the importance of trust in driving the acceptance of CT apps, but the approach to measuring trust and the trust referent under examination varies. For example, studies have found that high trust in the national government, the health care system, and science positively impacted willingness to use CT apps in Switzerland [14]. A US-based study found that trust in COVID-19 information positively influenced citizens’ comfort with and acceptance of CT [8]. In this study, our emphasis is on technology-related trust, as opposed to trust in an individual or organization. Specifically, we examined trust perceptions regarding a specific technology, namely a CT app. In addition, we investigated the perceptual factors that shape trust, namely perceptions of government motivations, need for surveillance, and propensity to trust technology (PTTT).

Second, studies have investigated the influence of privacy on CT adoption, with many studies finding that privacy concerns reduce intentions toward adopt CT apps [11,14]. Several studies conclude that privacy represents a barrier to the success of CT apps, with respondents in several countries citing privacy concerns as a reason for not installing apps [9] or conversely, those with low privacy concerns are more likely to use CT apps [12]. However, the influence of privacy concerns on CT adoption intentions was weak in another study [15]. Although privacy concerns are the most common proxy for measuring privacy across many contexts, the negative connotation and failure to directly capture privacy suggest the need for more precise operationalization [16]. Thus, we focus on perceived privacy defined as “an individual’s self-assessed state in which external agents have limited access to information about him or her” [16]. In this study, perceived privacy refers to a citizen’s belief in the level of privacy afforded by the CT app. Perceived privacy influenced intentions toward CT apps in a recent Brazilian study, thus supporting its use [17]. To further our understanding of perceived privacy, this study investigated the role of trust, perceived control, and perceived intrusion in shaping citizens’ perceptions of privacy.

Third, many CT studies are cross-sectional in nature, with the exception of a small number of longitudinal studies [12]. In addition, the literature focuses largely on whether citizens adopt CT apps or engage in behaviors recommended by CT apps such as staying at home [14,18]. This study broadens our understanding of citizens’ acceptance of CT apps by examining 2 variables related to acceptance, namely intention to adopt or continue using the app and willingness to disclose personal information. These acceptance variables are measured before and after the app launch, thereby deepening our understanding of how privacy and trust influence intentions toward and use of CT apps.

We argue that understanding the determinants of success of CT apps is critical not only for future digital CT but also for other contexts that require rapid digital technology adoption by the population [19]. This paper proceeds with an overview of the hypothesized relationships and our research context. Our methodology, data analysis, and results are presented in the following sections. The *Discussion* section outlines the implications of this study. The paper concludes with the limitations and avenues for future research.

### Theory

#### Overview

Privacy Calculus Theory (PCT) posits that before engaging in a behavior such as adopting a new technology or disclosing personal information, individuals will conduct a cognitive comparison of the costs and benefits associated with this behavior [20]. Individuals are likely to engage in the behavior for as long as the benefits outweigh the costs [20]. Thus, PCT has direct comparisons with the concept of calculus-based trust, which underpins trust decisions when engaging with new or unfamiliar trust referents [21]. PCT has been operationalized in many contexts using a variety of belief-based variables that can be grouped into confidence beliefs and risk or privacy beliefs. An extension of PCT that holds considerable promise for understanding privacy and trust in the context of CT apps is the inclusion of the procedural fairness theory. Procedural fairness refers to an individual’s perception that a particular activity is conducted fairly [22]. In the context of information privacy, fairness refers to the perception that personal data are collected and used fairly. Culnan and Armstrong [22] proposed that perceptions of procedural fairness can help citizens to “strike a balance between the competing forces of privacy and information use.” Individuals’ perceptions of the fairness of an organization’s data collection and use practices can influence their decision-making related to technology use and information disclosure [23]. In this study, we investigated the drivers of trust and privacy through the procedural fairness lens, which suggests the importance of factors related to the legitimacy of data collection (ie, the need for government surveillance and perceptions of the government’s motive for the app), the costs to the citizen (ie, perceived intrusion), and the level of autonomy and input citizens are afforded (ie, perceived control). Furthermore, the wider literature on fairness and trust suggests that individual differences in citizens’ PTTT are likely to play an important role alongside procedural fairness perceptions in trust [24], particularly given the unfamiliar referent of the CT app. In addition, the theory of reasoned action (TRA) allows us to consider the influence of these perceptions on behavioral outcomes. The TRA argues that individuals’ behaviors are determined by their beliefs, attitudes, and intentions [25]. We propose that individuals will express positive intentions toward downloading the app and disclosing information if they believe that the app demonstrates fairness.

#### Hypotheses

Trust is an important factor in the success of CT apps as demonstrated in recent studies [14]. Indeed, trust allows individuals to overcome concerns about uncertainty and fosters a willingness to engage in trust-related behaviors, such as disclosing information and engaging with technology [26]. In this study, our emphasis is on technology-related trust, which refers to individuals’ beliefs that the technology in question will perform as expected [27]. Trust perceptions refer to the extent to which the CT app will consistently deliver the proposed services and act in citizens’ best interest.

A significant body of theoretical work suggests that variables related to trust propensity are important drivers of trust perceptions [28], particularly in new and unfamiliar trust referents [29]. PTTT refers to a general tendency that is not specific to one trustee or situation but focuses on individuals’ willingness to depend on technology across different contexts and technologies [27]. We propose that, in the current context, PTTT will positively influence trust in CT apps.

- *Hypothesis 1a: PTTT will have a positive association with trust in the app.*

Surveillance programs are often introduced following large-scale events such as terrorist attacks [30]. Given the public health emergency caused by the COVID-19 pandemic, the importance of surveillance technologies, such as CT apps, is clear. The link between surveillance and trust has long attracted discussion with Dutton et al [31], highlighting the existence of *trust tension* between the government’s need to collect surveillance data and citizens’ concerns about the excessive use of this information. They assert that developing trust is imperative for resolving this tension. As CT apps require the surveillance of large groups of people [32], citizens must understand the need for government surveillance in the general sense to build trust in a CT app. Need for government surveillance refers to individuals’ perceptions that the government requires authority to access personal information using web-based means [33].

- *Hypothesis 1b: need for surveillance will have a positive association with trust in the app.*

Procedural fairness theory suggests that if individuals believe that the government’s motivations to introduce the app are rooted in good intentions, such as reducing virus transmission, they will express higher trust in the app to perform consistently and with their best interests in mind. Indeed, a perception of benevolent motives is at the heart of theories regarding trustworthiness [28], and perceptions of trustworthiness at the government level are likely to trickle down to influence trust in related referents [34]. Accordingly, we posit that government motive will engender trust in the app.

- *Hypothesis 1c: government motive will have a positive association with trust in the app.*

Government surveillance technologies can garner negative opinions, which may lead individuals to alter their behaviors. Perceived intrusion is described as a “harmful incursion into the personal information space” [35]. This relates to procedural justice and individuals’ perceptions of whether data are collected fairly in the CT app. Government surveillance technologies can be viewed as intrusive, but not all intrusions are considered harmful [35]. For example, the CT app may be viewed as intrusive, but as data are collected to reduce virus transmission, some individuals may not view this as harmful. Thus, only if individuals believe that the intrusiveness of the app is harmful to them, then their trust in the app is likely to be reduced.

- *Hypothesis 1d: perceived intrusion will have a negative association with trust in the app.*

Studies have asserted that privacy concerns represent a barrier to the success of CT apps [14,36]. However, there is a lack of research examining *if* citizens are likely to accept CT apps when they perceive that they provide some level of privacy. As perceived privacy refers to a perception that access to personal information by external agents is limited, the relevance of perceived intrusion as a privacy cost resulting from the use of an app is apparent. Indeed, the potential of CT apps to violate citizens’ privacy has been raised [36]. Thus, if individuals believe that the app is intrusive in a harmful manner in their informational space, they are less likely to believe that the app affords them privacy.

- *Hypothesis 2a: perceived intrusion will have a negative association with perceived privacy.*

Perceived control is described as individuals’ perceptions of their ability to control their personal information [37]. If individuals perceive that they maintain control over their information when transacting with a technology, they are more likely to feel comfortable that the technology will not act in a harmful manner [37], strengthening their perception of privacy in that context. A recent study supports the positive association between perceived control and perceived privacy of CT apps in Brazil [17]. We proposed that if individuals believe that they maintain control in the app, they will express higher levels of perceived privacy.

- *Hypothesis 2b: perceived control will have a positive association with perceived privacy.*

Finally, we argue that from a theoretical perspective, trust in the app will act as an uncertainty-reducing mechanism [38,39] and a heuristic that allows citizens to form privacy perceptions. Specifically, if citizens believe that the app will perform consistently and with their best interests in mind when using personal information, they will believe that the app provides some degree of privacy. This uncertainty reduction provides a foundation for facilitating other judgments of a technological artifact. Thus, trust in the app may influence perceptions of privacy.

- *Hypothesis 2c: trust in the app will have a positive association with perceived privacy.*

The success of CT apps is largely dependent on a critical mass of people downloading [40] and disclosing personal information. Thus, we draw on the TRA to consider 2 context-critical dependent variables: adoption intention and willingness to disclose personal information. Before the app launch, these variables were behavioral intentions. Adoption intention is described as an individual’s internal subjective judgment of the probability that they will perform the behavior in question [25]. The willingness to disclose information is based on an individual’s willingness to provide personal information when using the app [33]. Trust has repeatedly been identified as a driver of behavioral intentions across a range of contexts, including the acceptance of e-government technologies [41] and surveillance [30]. Indeed, behavioral operationalizations of trust often use behavioral intentions related to disclosure and reliance [42]. Empirical evidence suggests that perceived privacy influences intentions toward the use and data disclosure in general CT apps [17]. Although our study focuses on the privacy perception of a government-led CT app as opposed to general CT apps, we argue for similar effects. Finally, if individuals express high intentions toward downloading the app, we argue that they will be more willing to disclose personal information, as it is crucial to the app’s functionality.

- *H3a-b: trust in the app before the launch (a) and perceived privacy (b) will have a positive association with adoption intentions before the launch.*
- *H4a-c: trust in the app before the launch (a), perceived privacy (b), and adoption intentions before the launch (c) will have a positive association with disclosure intentions before the launch.*

There have been calls for research to understand the perceptions of a technology before and after the launch [43]. In the context of CT apps, it is important to explore how both perceptions of privacy and trust influence individuals’ behavioral intentions after launch. Thus, we examined both intentions after the launch. As some individuals may have already downloaded the app, adoption intentions are represented by future use intentions, which encompasses intentions to continue use among app users and intentions to adopt in the future among nonusers. TRA asserts that intentions will lead to behavior [44]. In other words, individuals’ intentions to download the app before the launch will be positively related to their use intentions after the launch. We draw on the TRA to posit effects similar to those hypothesized for before the launch. We argue that trust perceptions regarding the app and perceived privacy will positively impact use intentions after the launch.

- *H5a-b: trust in the app (a) and perceived privacy (b) will have a positive association with intentions to use after the launch.*
- *H5c: adoption intentions before the launch will have a positive association with intentions to use after the launch.*

The influence of trust and privacy on the willingness to disclose a specific surveillance technology after the launch has not been explored. Again, we leverage the TRA intention-behavior link and our hypotheses before the launch and argue that trust and perceived privacy will positively impact willingness to disclose personal information after the launch. Finally, as was the case before the launch, we argue that if individuals express high intentions toward using the app after the launch, they will be more willing to disclose personal information.

- *H6a-b: trust in the app (a) and perceived privacy (b) will have a positive association with disclosure intentions after the launch.*
- *H6c: intentions to use after the launch will have a positive association with disclosure intentions after the launch.*

**Figure 1:**
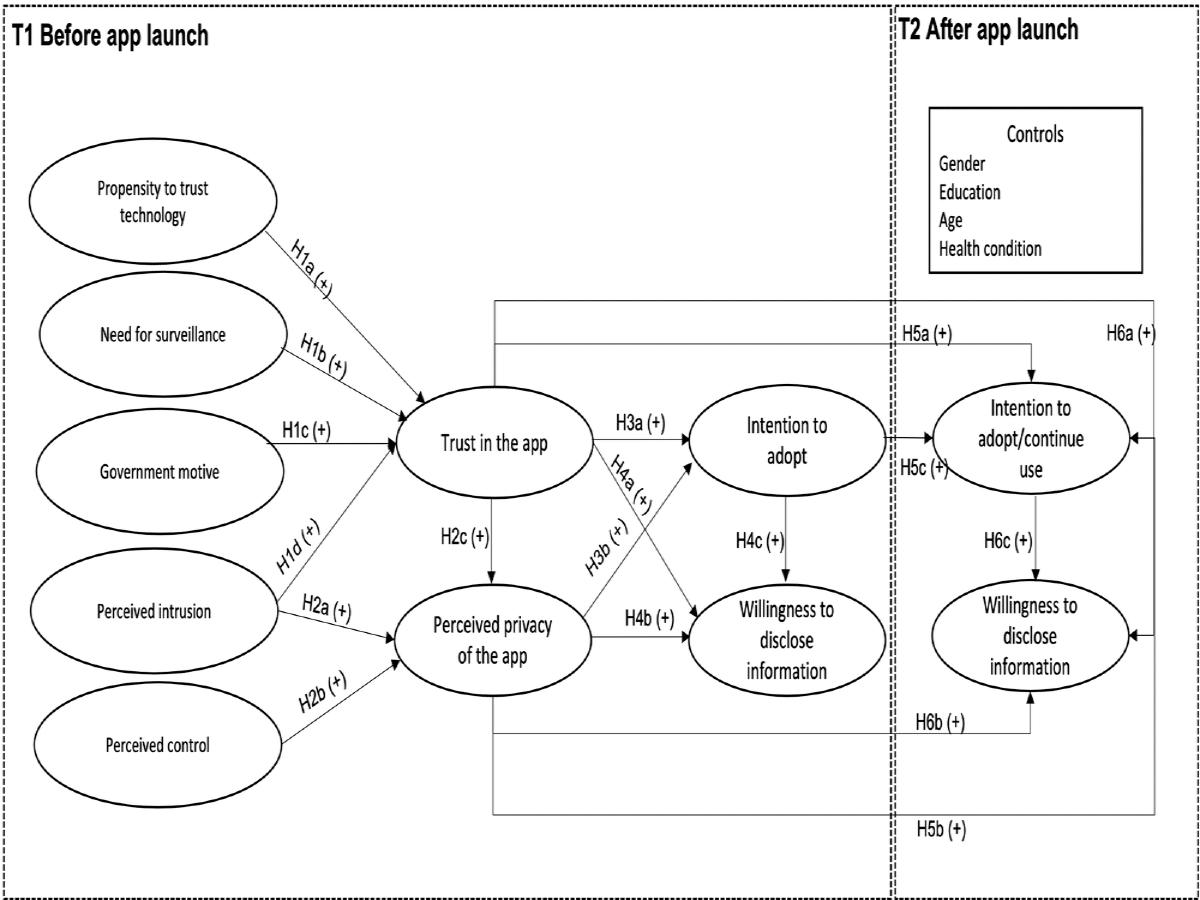
Research model.

The hypotheses are depicted in Figure 1.

## Methods

### Study Context

On July 6, 2020, the COVID Tracker app was launched in Ireland. COVID Tracker has 3 main features. CT uses Bluetooth and anonymous ID numbers to log phones within close contact for more than 15 minutes. It downloads the anonymous ID numbers of people who have tested positive and provides an alert if the user has been in close contact with those ID numbers. Check-in allows users to check for symptoms and seek health advice. The updates provide an overview of the daily COVID-19 figures. Within 48 hours of launch, 1 million people had downloaded the app [45]. By November 2021, the COVID Tracker app had over 1.7 million active users, representing 67% to 76% of the total possible Irish users [46].

### Instrument Development

We used existing scales when developing our instrument with minor wording amendments to adapt the items to the context. We provide the full list of items in Table S1 in Multimedia Appendix 1. The survey at T1 included general constructs related to PTTT, the need for government surveillance, and situationally framed constructs related to the proposed app, namely government motive for introducing the app, trust in the app, perceived surveillance in the app, perceived control in the app, and perceived privacy in the app. The dependent variables included intention to download the app on launch and willingness to disclose personal information to use the app. At T2, the emphasis was on future behavioral intentions, namely the use of the app and information disclosure. At T2, we asked participants if they had downloaded the app, and app users’ intentions to continue to use the app were examined and nonusers’ intentions to download the app in the future were examined. These intentions were combined as use intentions for analysis. Willingness to disclose personal information to the app was examined across both users and nonusers of the app. In addition, gender, age, and education were used as the control variables. Respondents were asked if they had any health condition that left them particularly susceptible to COVID-19. This was also a control variable. Both surveys were piloted and tested among a small panel of survey design experiments, and several wording amendments were made. Respondents were asked to answer demographic and health questions first, followed by general perceptual constructs and control variables, the order of which was randomized. In the third section, participants were presented with a neutrally framed description of the proposed national CT app at T1, and a description of the launched app was presented at T2. The final section examined perceptions of the app, behavioral intentions, and willingness to disclose personal data, the order of which was randomized.

### Ethical Considerations

Ethics approval was obtained from the university’s research ethics committee before the launch of the survey (DCUREC/2020/096).

### Recruitment

Qualtrics (Qualtrics International Inc) was used to host and administer the survey using their panel services. An attention check was included to screen for unengaged responses. A total of 1109 complete responses were received at T1 and were recontacted at T2. After 2 follow-up invitations, 405 responses were received at T2, achieving a response rate of 36.5%. Responses at T1 and T2 were, on average, 77 days apart. Incomplete responses and responses failing the attention check were removed using Qualtrics. The sample characteristics are illustrated in Table 1, along with the population characteristics as per the latest census at the time of data collection. Overall, the sample demographics were similar to the population characteristics of Ireland, as reported in the last census, and included respondents from the 26 counties within the country. Of the 405 respondents in T2, 202 had downloaded the app and 203 had not yet downloaded the app.

**Table 1:** Sample and population characteristics (N=405).

		Sample, n (%)	Population (%)^a,b^
**Gender**			
	Man	180 (44.4)	49
	Woman	225 (55.6)	51
	Rather not say	0 (0)	N/A^c^
**Age group (years)**			
	18-24	13 (3.2)	11
	25-44	124 (30.6)	39
	45-64	173 (42.7)	32
	≥65	95 (23.5)	18
**Employment**			
	Employed	186 (45.9)	45
	Self-employed	26 (6.4)	8
	Unemployed	36 (8.9)	6
	Student	11 (2.7)	11
	Unavailable for work	42 (10.4)	12
	Retired	104 (25.7)	15
**Education**			
	Secondary school	157 (38.8)	28
	Trade	5 (1.2)	7
	Diploma	32 (7.9)	12
	Bachelor degree	133 (32.8)	27
	Other qualification	64 (15.8)	14
	Doctorate degree	14 (3.5)	1

^a^Population figures are based on data provided by the Irish Central Statistics Office in the latest population census at the time of data collection (ie, 2016).

^b^Employment and education figures include all people aged ≥15 years living in Ireland in 2016, whereas our sample only includes people aged ≥18 years.

^c^N/A: not applicable.

## Results

### Reliability and Validity Testing

Data analysis was performed using IBM AMOS (version 25.0). The proposed model comprising 11 constructs was examined using Confirmatory Factor Analysis with further detail provided in Table S2 in Multimedia Appendix 1. In total, 3 items were dropped from the PTTT because of their low loadings. The model indicated a good fit: c_min_/df=1.805, comparative fit index=0.980, root mean square error of approximation=0.045, and standardized root mean squared residual=0.034. A test of equal specific bias was conducted to examine potential common method bias among the data [47]. This test demonstrated an unevenly distributed bias; thus, the specific bias construct was retained for causal analysis to control for any effects because of method [48]. The validity and reliability of all the constructs were explored. Convergent validity was assessed by calculating the average variance extracted (AVE). As all the variables had AVE scores above 0.500, convergent validity was achieved [49]. Discriminant validity was tested by comparing the square root of the AVE with the interconstruct correlations. As the square root of AVE was higher than the interconstruct correlations, discriminant validity was achieved, as shown by the italicized diagonal values in Table 2. Reliability was assessed by calculating composite reliability for each construct. With composite reliability scores above 0.700, all constructs were reliable [50]. Further details on the validity testing are provided in the supplementary appendices available on the web.

**Table 2:** Validity and reliability statistics.

	Composite reliability	Average variance extracted	1	2	3	4	5	6	7	8	9	10	11
Need for Surveillance	0.868	0.526	*0.725* ^a^	—^b^	—	—	—	—	—	—	—	—	—
Propensity to trust technology	0.870	0.626	0.183^c^	*0.791*	—	—	—	—	—	—	—	—	—
Perceived control	0.967	0.879	0.329^d^	0.210^d^	*0.938*	—	—	—	—	—	—	—	—
Willingness to disclose information (T1)	0.983	0.966	0.378^d^	0.271^d^	0.682^d^	*0.983*	—	—	—	—	—	—	—
Intention to adopt (T1)	0.990	0.970	0.342^d^	0.270^d^	0.666^d^	0.872^d^	*0.985*	—	—	—	—	—	—
Perceived intrusion	0.932	0.820	−0.181^e^	−0.109^e^	−0.471^d^	−0.508^d^	−0.397^d^	*0.906*	—	—	—	—	—
Trust in App	0.916	0.786	0.365^d^	0.336^d^	0.702^d^	0.814^d^	0.784^d^	−0.491^d^	*0.886*	—	—	—	—
Perceived Privacy in App	0.962	0.895	0.371^d^	0.259^d^	0.852^d^	0.801^d^	0.753^d^	−0.603^d^	0.852^d^	*0.946*	—	—	—
Intention to adopt or use (T2)	0.991	0.973	0.234^d^	0.167^e^	0.500^d^	0.612^d^	0.658^d^	−0.347^d^	0.574^d^	0.548^d^	*0.986*	—	—
Willingness to disclose information (T2)	0.985	0.970	0.309^d^	0.241^d^	0.537^d^	0.678^d^	0.666^d^	−0.401^d^	0.686^d^	0.646^d^	0.844^d^	*0.985*	—
Government motive	0.971	0.894	0.246^d^	0.332^d^	0.550^d^	0.682^d^	0.654^d^	−0.378^d^	0.799^d^	0.658^d^	0.494^d^	0.575^d^	*0.946*

^a^As the square root of AVE was higher than the interconstruct correlations, discriminant validity was achieved, as shown by the italicized values.

^b^Not available.

^c^Significance at 10% level.

^d^Significance at 1% level.

^e^Significance at 5% level.

### Hypotheses Testing

The causal model was tested using Structural Equation Modeling in AMOS. The model indicated a good fit c_min_/df=2.835, comparative fit index=0.985, root mean square error of approximation=0.067, and standardized root mean squared residual=0.021. H1a-d focused on the antecedents of trust in the app. H1a proposed a positive relationship between PTTT and trust. The data revealed a positive, significant relationship supporting hypothesis 1a (β=.074; *P*=.006). Hypothesis 1b posited that the perceived need for government surveillance would positively influence trust. This was also supported (hypothesis 1b: β=.119; *P*<.001). H1c posited that government motive would be positively related to trust. The data supported the hypothesis (hypothesis 1c: β=.671; *P*<.001). The negative relationship between perceived intrusion and trust was supported (hypothesis 1d: β=−.224; *P*<.001). The following set of hypotheses examined the antecedents of perceived privacy. Hypothesis 2a proposed a negative association between perceived intrusion and perceived privacy. These data supported hypothesis 2a (β=−.165; *P*<.001). We hypothesized that perceived control and trust would be positively related to perceived privacy. Both relationships were supported (hypothesis 2b: β=.451; *P*<.001; H2c: β=.466; *P*<.001).

In terms of T1 behavioral intentions, it was posited that perceived privacy and trust would positively influence the intention to adopt. Both relationships were supported (hypothesis 3a: β=.247; *P*<.001; hypothesis 3b: β=.590; *P*<.001). H4a-c proposed that trust, perceived privacy, and adoption intentions would positively influence willingness to disclose personal information. The data revealed that trust (β=.215; *P*<.001), perceived privacy (β=.208; *P*<.001), and adoption intentions (β=.550; *P*<.001) each positively influenced willingness to disclose. In terms of T2 behavioral intentions, hypothesis 5a to 5c proposed that trust, perceived privacy, and adoption intentions (T1) would all influence use intentions (T2). Both trust (β=.124; *P*=.15) and perceived privacy (β=.042; *P*=.60) had a positive but nonsignificant influence on intentions. T1 adoption intentions significantly influenced use intentions supporting hypothesis 5c (β=.530; *P*<.001). Finally, trust, perceived privacy, and use intentions at T2 were proposed to positively influence the willingness to disclose at T2. Perceived privacy (β=.042; *P*=.40) had a nonsignificant influence, whereas trust and use intentions had significant relationships supporting hypothesis 6a and hypothesis 6c (hypothesis 6a: β=.250; *P*<.001; H6c: β=.655; *P*<.001). In terms of control variables, COVID-19 vulnerable illness had a significant negative effect on individuals’ willingness to disclose at T1 (β=−.043; *P*=.009), and education had a positive effect on T2 use intentions (β=.075; *P*=.04).

The model explains 74.4% of variance in trust, 91% of variance in perceived privacy, 66.6% of variance in T1 adoption intentions, 45.9% of variance in T2 adoption intentions, and 83.9% and 79.4% of variance in willingness to disclose at T1 and T2. Bootstrapping using 2000 samples and a confidence level of 90% was conducted in AMOS to explore the indirect effects. The findings revealed that perceived privacy had a significant influence on T2 adoption intentions (β=.131; *P*=.001) and willingness to disclose at T2 (β=.127; *P*=.04). Similarly, trust had a significant influence on intention to download (β=.394; *P*<.001) and willingness to disclose at T2 (β=.386; *P*<.001). Further detail is provided in Table S3 in Multimedia Appendix 1.

## Discussion

### Principal Findings

This study focuses on understanding how citizens’ beliefs shape their perceptions of privacy and trust to influence their acceptance of a CT app for COVID-19. Our study found that trust in the app was positively influenced by the PTTT, perceived government motive, and perceived need for government surveillance, whereas perceived intrusion had a negative influence. Perceived privacy was positively shaped by perceptions of control and trust and negatively shaped by perceived invasion. The study examined citizens’ acceptance of CT app at 2 time intervals. Before launch, the intention to adopt the app was positively influenced by trust and perceived privacy, and willingness to disclose personal information to the app was influenced by adoption intentions, trust, and perceived privacy. However, postlaunch use intentions were influenced only by prelaunch adoption intentions, whereas willingness to disclose personal information was influenced by trust and postlaunch use intentions but not by perceived privacy. Although the insignificant results may suggest that perceived privacy is only important before launch, and the influence of trust on use intentions diminishes over time, post hoc bootstrapping analysis revealed that both perceived privacy and trust had significant indirect relationships with use intentions and willingness to disclose information at T2. This suggests that both perceptions play a role in influencing behavioral intentions before and after the launch.

### Contributions

Studies have shown that trust and privacy are important factors in the success of health surveillance technologies such as CT apps [11]. Our study leverages the procedural fairness theory to understand how citizens’ perceptions of trust and privacy emerge in the context of a CT app. This context is interesting, as the technology in question was introduced by the national government and backed by several organizations with the app’s potential benefits extending to the public at large. Therefore, it is important to look beyond the role of a single organization in driving perceptions of fairness to consider a broader set of antecedents that drive perceptions of trust and privacy in this context. Indeed, as research has shown the importance of trust and privacy in the success of mHealth and health technologies introduced by health care organizations and indeed national health systems [51], our study contributes to the broader health technology literature.

The first contribution of our study is the deeper understanding of how trust is formed in this context. Lack of trust in the government has been identified as a barrier to CT app adoption [18]. Thus, it is important to provide governments and public health organizations with insights into how trust in CT can be fostered [8]. Our findings bolster assertions regarding the important role of fairness perceptions and suggest that citizens’ trust perceptions regarding the app are formed based on their beliefs about the legitimacy of data collection, perceived autonomy, and perceived costs. Legitimacy is represented by citizens’ perceptions of the need for government surveillance and perceptions of the government’s motive for introducing the app, autonomy is captured by perceptions of control over one’s information in the app, and perceived costs to the individual relate to perceptions of personal intrusion.

The second contribution of our study is the investigation of how perceptions of privacy are formed. Many studies have highlighted the negative impact of privacy concerns on CT adoption [11,52-54]. However, we argue that privacy can be seen as a factor driving adoption if citizens believe that apps can afford them with some level of privacy. Our findings demonstrate that citizens’ privacy perceptions are shaped by trust in the app, which encompasses legitimacy perceptions and their perception of control offered by the app, and are negatively influenced by perceptions of intrusion. By highlighting the importance of fairness perceptions and elucidating the role of several perceptual factors at the governmental level (need for surveillance and government motives) and the app level (perceived intrusion and perceived control), which have been sparingly studied to date, our study advances our understanding of how privacy perceptions are developed in this context.

Understanding the factors driving CT app acceptance is paramount for future outbreaks [8]. The third contribution relates to understanding how citizens form intentions toward CT apps at different stages of the implementation process. Our study provides support for the influence of perceived privacy on individuals’ intentions to adopt an app and willingness to disclose personal information before launch and support for an indirect influence over time on future use and willingness to disclose data. This finding supports many studies that found that privacy concerns negatively impacted adoption intentions toward mHealth [51]. In the context of a national CT app, if individuals perceive that the app offers a sufficient state of privacy, they will express positive intentions toward adoption and information disclosure before and after the app launch. Put simply, perceived privacy can have a sustained positive influence on behavioral intentions.

Trust has been widely studied within the privacy and other domains to understand individuals’ intentions to disclose information [42]. Trust in the app was found to influence individuals’ adoption and disclosure intentions before launch, indirectly influencing use intentions after launch and directly influencing disclosure intentions after launch. These findings suggest that the influence of initial trust perceptions prevails over time and may operate as a heuristic for interacting with the app on an ongoing basis. The stability of trust perceptions and their ongoing influence are a relatively nascent topic, although some theorists have suggested the possibility of trust as a heuristic [55]. Our research provides empirical evidence for this phenomenon and offers further support for claims that initial trust perceptions might be relatively robust and long acting [56].

### Implications for Practice

The findings of this study have several practical implications. First, the trust tension between public good and the intrusiveness of surveillance technologies has led researchers to emphasize the importance of effective trust-building strategies when introducing surveillance programs [31]. Indeed, citizens in the United States and Germany have expressed concerns regarding possible surveillance stemming from CT apps [9]. Our study shows that citizens’ perceptions of trust and privacy can be influenced by fairness perceptions based on their beliefs regarding the need for surveillance and the government’s motives for introducing surveillance technologies, the perceived control they are offered over their personal information and negatively influenced by their perceptions of the intrusiveness of these technologies. Thus, governments should focus on transparency in their public health surveillance efforts, including the involvement of data protection authorities and civil liberties advocates throughout the project life cycle, potentially through a privacy advisory committee [57]. This transparency should be extended to communication with citizens on the need and purpose of a technology while stressing the control they have over their personal information. Our research suggests that early communications that shape first impressions are particularly important. Such practices not only comply with data protection laws, such as the EU (European Union) General Data Protection Regulation, but also foster a sense of trust and ultimately influence the use of technology.

Second, our findings highlight the positive influence of privacy perceptions on adoption and disclosure. Thus, we argue that privacy should not be viewed as a barrier to new technologies, such as mHealth or CT apps, but rather as an important consideration throughout the design, implementation, and postlaunch stages. Designers should ensure compliance with the regulatory requirements for consent and control. Governments and other organizations charged with introducing new technologies should ensure that they clearly communicate their compliance with regulations and the considerations of individuals’ personal data. Given that CT apps provide data on the location, copresence, and potentially ePHI of not only the focal person but also others that they have been in contact with, the principles of both necessity and proportionality would appear to be key. As per Ienca and Vayena [4], data collection must (1) be proportional to the seriousness of the public health threat, (2) be limited to what is necessary to achieve a specific public health objective, and (3) be scientifically justified. Policy makers and public health decision makers need to consider what communication and control mechanisms can be introduced to (1) build trust with the public and (2) repair trust, if necessary. This includes declaring what data will be collected and used while the app is live and by whom, confirming that data have been deleted, when no longer relevant (as is the case with COVID-19 data) or once the app is no longer required.

Third, in the context of technologies that require mass acceptance and willingness to share personal data, the focus cannot be placed solely on the number of downloads but must account for actual use and disclosure behaviors. Individuals’ intentions to download CT apps influence their willingness to disclose personal information both before and after launch. Once they have downloaded the app, it is critical that decision makers encourage use and that the widespread use of the app is linked, through public communication, to successful intervention strategies so that the benefits to the individual and society are reinforced.

### Limitations and Directions for Future Research

This study has several limitations. First, other factors may influence privacy and trust perceptions or moderate the relationships between trust and privacy and adoption. Although it is not possible to consider all potential antecedents and intervening variables, it would be interesting to explore the role of other prominent perceptions, such as perceived sensitivity, as these apps require users to disclose identifying information, health information, and location information, all of which are arguably sensitive. Second, although our study considers 2 important technology use outcomes, before and after the launch, this approach has limitations. First, the collection of data from the same respondents at multiple time points inevitably led to a drop in responses. Although we sent repeated invitations during the second phase of data collection, the final sample that completed both surveys was smaller. Although this is commonplace within this approach and our sample characteristics are similar to the broader population of Ireland, we acknowledge that a large sample would be ideal and stress the importance of considering the sample size when drawing inferences from our study. Second, our 2 time points did not allow us to take full advantage of the potential to model longitudinal change trajectories over time. Further work is needed to incorporate time more fully into our understanding of how privacy and trust influence adoption and use behaviors.

Third, our study relies on individuals’ self-reported adoption and disclosure intentions. This approach is commonplace in the privacy and technology adoption literature streams, and it would not have been possible to study actual behaviors. However, we must acknowledge that intentions are not always matched with behaviors and that information disclosure is not always accurate or true. In other settings, it may be more feasible to collect more objective behavioral data, and we would encourage researchers to do so, particularly in settings where widespread adoption is required for success. In addition, studies may go beyond our focus to understand disclosure behaviors at a deeper level and examine privacy-protective behaviors, such as withholding information or falsifying information. These protective behaviors are potentially dangerous in contexts such as CT apps because of the reliance on accurate data to track virus transmission.

Finally, our study explores a public health surveillance context where the focal person volunteers to participate and therefore has notice of the surveillance, control of their data and gives explicit consent. There are several conditions under which public health surveillance, including name reporting, may be undertaken without notice or explicit patient consent with well-established justifications in public health ethics, science, and law [58]. Even in the context of COVID-19, digital CT has not always been voluntary. In China, there is evidence of digital CT without notice or consent [59]. Furthermore, even when the focal person has notice and gives consent, contacts of the focal person have not given explicit consent. Although the primary focal person is subject to direct active surveillance, the secondary focal person is subject to passive indirect surveillance. In addition to the ethical issues that such practices raise, particularly where there is coordination and data exchange between private firms and the government [60], testing the theoretical framework developed in this study in this new context may provide a fruitful avenue of research. Similarly, aggregated anonymous spatiotemporal data sourced from commercial providers have been used as proxies for human movement and social interaction and as indicators of the effectiveness of social distancing interventions [61]. Although these data are currently anonymous, governments have already mandated access to identifiable data on the basis that the public interest overrides privacy rights [62]. This context may provide interesting insights and further extend our understanding of the limits of consumer acceptance of governmental health surveillance.

### Conclusions

The COVID-19 pandemic was the first time governments implemented large-scale digital CT. Its success as a public health intervention depended on rapid technology adoption by a significant proportion of the population. Here, surveillance is active, and the target of government surveillance through COVID-19 digital CT apps is an active participant in sharing data with the government on their personal health status, their location, and often their social network. The opportunity to study such an empirical context is not only rare but also the time frame for research is limited. Understanding the formation of individuals’ perceptions of trust and privacy in this context and how these perceptions influence their acceptance of digital CT apps is critical not only for informing the design of future digital CT initiatives but also for other situations that require rapid digital technology adoption by a significant proportion of society. If governments wish to leverage the power of digital technologies to control future public health threats, we recommend 3 principles to guide the design of both their surveillance initiatives and communications with the public—necessity, transparency, and proportionality—before and after the launch.

## Appendix

Multimedia Appendix 1 Survey items, validity, and reliability testing.

## Abbreviations

AVE: average variance extracted
CT: contact tracing
ePHI: electronic personal health information
EU: European Union
mHealth: mobile health
PCT: Privacy Calculus Theory
PTTT: propensity to trust technology
TRA: theory of reasoned action
