# Supplementary material for: Investigating Citizens’ Acceptance of Contact Tracing Apps: Quantitative Study of the Role of Trust and Privacy
Source: JMIR Mhealth Uhealth. 2024 Jan 18;12:e48700. doi: 10.2196/48700 (PMC10835590; doi:10.2196/48700)
Supplement: Multimedia Appendix 1 [file mhealth_v12i1e48700_app1.pdf]

APPENDIX A: Instrumentation

The final items for all constructs are illustrated below in Table A.1

Table A.1 Final constructs and items		
**Construct**	**Item**	**Wording**
Propensity to Trust Technology (7 point agreement scale McKnight et al. 2011)	PTTT1	I believe that most technologies are effective at what they are designed to do.
	PTTT 2	A large majority of technologies are excellent.
	PTTT 3	Most technologies have the features needed for their domain.
	PTTT 4	I think most technologies enable me to do what I need to do.
Need for Government Surveillance (7 point agreement scale adapted from Dinev et al., 2008	PNGS1	The government needs to have greater access to personal information
	PNGS 2	The government needs to have greater access to individual bank accounts
	PNGS 3	The government needs broader wiretapping authority
	PNGS 4	The government needs to have more authority to use high tech surveillance tools for Internet eavesdropping
Perceptions of Government Motivations (7 point agreement scale based on Grant, 2008)	Why do you think the government introduced this app?	
	GM1	Because they care about benefiting others through the app
	GM2	Because they want to help others through the app
	GM 3	Because they want to have a positive impact on others
	GM 4	Because it is important for them to do good others through my work
Perceived Control (7 point agreement scale adapted from Dinev et al. 2013)	PCIA1	I think I have control over what personal information is released by the app.
	PCIA 2	I believe I have control over how personal information is used by the app.
	PCIA 3	I believe I have control over what personal information is collected the app.
	PCIA 4	I believe I control my personal information provided to the app.
Trust in App (7 point agreement scale adapted from Venkatesh et al. 2016)	TIA1	I believe that the app acts in my best interest.
	TIA2	I expect the app to be sincere and genuine.
	TIA3	I believe that the app performs its role very well

Perceived intrusion in App (7 point agreement scale adapted from Xu et al. 2008)	PIIA1	I feel that as a result of using the app, others would know about me more than I am comfortable with.
	PIIA2	I believe that as a result of using app, information about me that I consider private would be more readily available to others than I would want.
	PIIA3	I feel that as a result of using the app information about me would get out there that, if used, will invade my privacy.
Perceived Privacy (7 point agreement scale adapted from Dinev et al. 2013)	PPIA1	I feel I would have enough privacy when using the app.
	PPIA2	I am comfortable with the amount of privacy I will have when using the app.
	PPIA3	I think my online privacy would be preserved when I use the app.
Intention to adopt (T1) (7 point agreement scale adapted from Venkatesh et al., 2003)	INT1	I intend to download the app when it becomes available.
	INT2	I plan to download the app when it becomes available.
	INT3	I predict I will download the app when it becomes available.

**Table A.1 Final constructs and items (cont'd)**		
Willingness to disclose personal information to the app (T1) (7 point agreement scale based on Xu et al. 2009)	DIS1	I am willing to share my personal information, location data, and health information through this app
	DIS2	I am likely to share my personal information, location data, and health information through this app
Usage Intentions (T2) (7 point agreement scale based on Bhattacherjee, 2001 and Venkatesh et al., 2003)	USE1	I intend to download/keep using the app in the future.
	USE2	I plan to download/keep using the app in the future.
	USE3	I predict I will download/keep using the app in the future.
Willingness to disclose personal information to the app (T2) (7 point agreement scale based on Xu et al. 2009)	DIST2_1	I am willing to share my personal information, location data, and health information through this app
	DIST2_2	I am likely to share my personal information, location data, and health information through this app

APPENDIX B: VALIDITY TESTING AND ANALYSIS DETAILS

Establishing Validity

Prior to data analysis, convergent and discriminant validity were explored using the AMOS validity tool (Gaskin, James, Lim and Steed, 2023). Convergent validity requires items that should be related to each other such as items expected to converge on to reflect the same construct, to demonstrate high correlations with other (Straub, Boudreau & Gefen, 2004). Discriminant validity on the other hand requires that items which are not expected to be related to each demonstrate low correlations (Straub et al., 2004).

Convergent Validity

To assess convergent validity, the factor loadings of all items across each construct was examined. As shown in table B-1, all items with the exception of PNGS1 are above the .700 threshold for establishing convergent validity. In line with Kline et al. (2012), we also assess the AVE for each construct as shown in table B-2. As the AVE for each latent variable is above 0.50, convergent validity is established.

Discriminant Validity

To examine discriminant validity, we conducted the Fornell-Larcker test to investigate if the AVE for each construct was greater than interconstruct correlations (Fornell & Larcker, 1981). As the square root of the AVE for each construct is greater than any correlations between the construct and other constructs, discriminant validity is achieved. This is demonstrated in table B-3 on the diagonal and bold.

Assessing Multicollinearity

It is important to explore multicollinearity particularly in SEM models. To examine multicollinearity, we assessed the variance inflation factors (VIFs) using the stringent 5.0 threshold for reflective constructs (Petter et al., 2007). As shown in table B.5, the VIF for all constructs is below 5. Thus, multicollinearity does not represent an issue with this data.

Table B.1 Factor Loadings for All items

Construct	Item	Factor Loading	Mean	Std. Dev
Propensity to Trust Technology	PTTT1	.723	5.06	1.13
	PTTT 2	.864	5.41	1.10
	PTTT 3	.805	5.14	1.07
	PTTT 4	.767	5.45	0.99
Need for Government Surveillance	PNGS1	.697	2.76	1.49
	PNGS 2	.722	2.06	1.43
	PNGS 3	.832	2.57	1.67
	PNGS 4	.811	2.92	1.83

Perceptions of Government Motivations	GM1	.947	4.80	1.55
	GM2	.978	4.94	1.52
	GM 3	.968	4.94	1.53
	GM 4	.886	4.76	1.50
Perceived Control	PCIA1	.921	3.65	1.74
	PCIA2	.965	3.52	1.73
	PCIA3	.947	3.57	1.74
	PCIA4	.917	3.55	1.77
Trust in App	TIA1	.945	4.51	1.65
	TIA2	.863	4.86	1.66
	TIA3	.847	4.68	1.55
Perceived intrusion in App	PIIA1	.908	4.26	1.69
	PIIA2	.898	4.28	1.67
	PIIA3	.908	4.32	1.69
Perceived Privacy	PPIA1	.948	3.80	1.75
	PPIA2	.958	3.89	1.80
	PPIA3	.925	3.91	1.76
Intention to adopt (T1)	INT1	.989	4.45	1.94
	INT2	.990	4.45	1.92
	INT3	.974	4.44	1.96
Willingness to disclose personal information to the app (T1)	DIS1	.985	4.57	1.89
	DIS2	.979	4.51	1.93
Usage Intentions (T2)	USE1	.991	4.62	2.11
	USE2	.978	4.63	2.13
	USE3	.989	4.63	2.14
Willingness to disclose personal information to the app (T2)	DIST2_1	.985	4.50	1.92
	DIST2_2	.984	4.45	1.94

Table B.2 AVE for all Constructs	
**Construct**	**AVE**
Propensity to Trust Technology	.626
Need for Government Surveillance	.589
Perceptions of Government Motivations	.894
Perceived Control	.879
Trust in App	.786
Perceived intrusion in App	.820
Perceived Privacy	.890
Intention to adopt (T1)	.970
Willingness to disclose personal information to the app (T1)	.964
Usage Intentions (T2)	.973
Willingness to disclose personal information to the app (T2)	.970

Table B.3 Assessing Discriminant Validity

	CR	AVE	1	2	3	4	5	6	7	8	9	10	11
1. PNGS	.868	.526	**.725**										
2. PTTT	.870	.626	.183*	**.791**									
3. PCIA	.967	.879	.329***	.210***	**.938**								
4.DIS (T1)	.983	.966	.378***	.271***	.682***	**.983**							
5.INT (T1)	.990	.970	.342***	.270***	.666***	.872***	**.985**						
6. PIIA	.932	.820	-.181*	-.109*	-.471***	-.508***	-.397***	**.906**					
7.TIA	.916	.786	.365***	.336***	.702***	.814***	.784***	-.491***	**.886**				
8. PPIA	.962	.895	.371***	.259***	.852***	.801***	.753***	-.603***	.852***	**.946**			
9. USE (T2)	.991	.973	.234***	.167**	.500***	.612***	.658***	-.347***	.574***	.548***	**.986**		
10.DIS (T2)	.985	.970	.309***	.241***	.537***	.678***	.666***	-.401***	.686***	.646***	.844***	**.985**	
11. GM	.971	.894	.246***	.332***	.550***	.682***	.654***	-.378***	.799***	.658***	.494***	.575***	**.946**

Table B.4 Validity Analysis - Confidence Intervals						
	**CR**	**AVE**	**Lower 95% CR**	**Upper 95% CR**	**Lower 95% AVE**	**Upper 95% AVE**
**PNGS**	0.868	0.526	0.813	0.910	0.423	0.630
**PTTT**	0.870	0.626	0.811	0.914	0.520	0.726
**PCIA**	0.967	0.879	0.947	0.981	0.818	0.929
**DIS (T1)**	0.983	0.966	0.972	0.990	0.945	0.981
**INT (T1)**	0.990	0.970	0.982	0.995	0.948	0.985
**PIIA**	0.932	0.820	0.892	0.960	0.733	0.888
**TIA**	0.916	0.786	0.878	0.944	0.707	0.850
**PPIA**	0.962	0.895	0.945	0.975	0.852	0.929
**INT (T2)**	0.991	0.973	0.981	0.997	0.945	0.990
**DIS (T2)**	0.985	0.970	0.972	0.994	0.946	0.988
**GM**	0.971	0.894	0.957	0.981	0.849	0.929

Table B.5 Multicollinearity Statistics for all Constructs		
**Construct**	**Tolerance**	**VIF**
Propensity to Trust Technology	.867	1.153
Need for Government Surveillance	.881	1.135
Perceptions of Government Motivations	.398	2.515
Perceived Control	.884	1.131
Trust in App	.236	4.230
Perceived intrusion in App	.662	1.511
Perceived Privacy	.246	4.063
Intention to adopt (T1)	.241	4.516
Willingness to disclose personal information to the app (T1)	.292	3.424
Usage Intentions (T2)	.241	4.147

Willingness to disclose personal information to the app (T2)	.297	3.365

APPENDIX C. STRUCTURAL EQUATION MODEL RESULTS

Structural equation modelling (SEM) was conducted using AMOS v25. The results of the hypothesis testing are outlined below in Table C.1.

Table C.1 Structural model results

Hypothesis	*P*-value	Beta	Supported
**H1a.** PTTT → Trust	.006	0.074	Yes *P=.006*
**H1b.** PNGS→ Trust	.001	0.119	Yes *P<.001*
**H1c.** Gov. Motive Trust	.001	0.671	Yes *P<.001*
**H1d.** PIIA → Trust	.001	-0.224	Yes *P<.001*
**H2a.** PIIA → Per.Privacy	.001	-0.165	Yes *P<.001*
**H2b.** PCIA → Per.Privacy	.001	0.451	Yes *P<.001*
**H2c.** Trust → Per.Privacy	.001	0.466	Yes *P<.001*
**H3a.** Trust → INT (T1)	.001	0.590	Yes *P<.001*
**H3b.** Per.Privacy INT (T1)	.001	0.247	Yes *P<.001*
**H4a.** Trust → DIS (T1)	.001	0.215	Yes *P<.001*
**H4b.** PPIA → DIS (T1)	.001	0.208	Yes *P<.001*
**H4c.** INT (T1) → DIS (T1)	.001	0.550	Yes *P<.001*
**H5a.** Trust → USE (T2)	.152	**0.124**	**No *P>.050***
**H5b.** PPIA → USE (T2)	.596	**0.042**	**No *P>.050***
**H5c.** INT (T1) → USE (T2)	.001	0.550	Yes *P<.001*
**H6a.** Trust → DIS (T2)	.001	0.250	Yes *P<.001*
**H6b.** PPIA → DIS (T2)	**.404**	**0.042**	**No *P>.050***
**H6c.** USE (T2) → DIS (T2)	.001	0.655	Yes *P<.001*

Examining Indirect Effects

To examine indirect effects on post-launch use intentions and disclosure intentions, bootstrapping with 2,000 samples was conducted in AMOS v.25. The results are illustrated below in table C.2.

Table C.2 Standardized indirect effects

Hypothesis	*P*-value	Beta	Supported
Trust → USE (T2)	.001	0.394	Yes *P<.001*
Per.Privacy → USE (T2)	.002	0.386	Yes *P<.050*
Trust → DIS (T2)	.001	0.131	Yes *P<.001*
Per.Privacy → DIS (T2)	.036	0.127	Yes *P<.050*

References for Appendices

1. Mcknight DH, Carter M, Thatcher JB, Clay PF. Trust in a specific technology: An investigation of its components and measures. ACM Transactions on management information systems (TMIS). 2011 Jul 1;2(2):1-25
2. Dinev T, Hart P, Mullen MR. Internet privacy concerns and beliefs about government surveillance–An empirical investigation. The Journal of Strategic Information Systems. 2008 Sep 1;17(3):214-33.
3. Grant AM. Does intrinsic motivation fuel the prosocial fire? Motivational synergy in predicting persistence, performance, and productivity. Journal of applied psychology. 2008 Jan;93(1):48.
4. Dinev T, Xu H, Smith JH, Hart P. Information privacy and correlates: an empirical attempt to bridge and distinguish privacy-related concepts. European Journal of Information Systems. 2013 May 1;22(3):295-316.
5. Venkatesh V, Thong JY, Chan FK, Hu PJ. Managing citizens' uncertainty in e-government services: The mediating and moderating roles of transparency and trust. Information systems research. 2016 Mar;27(1):87-111.
6. Xu H, Dinev T, Smith HJ, Hart, P. Examining the formation of individual's privacy concerns: Toward an integrative view. Proceedings of the 29th International Conference on Information Systems; 2008 Dec 14-17; Paris, France. AIS Electronic Library (AISeL); 2008.
7. Venkatesh V, Morris MG, Davis GB, Davis FD. User acceptance of information technology: Toward a unified view. MIS quarterly. 2003 Sep 1:425-78.
8. Xu H, Teo HH, Tan BC, Agarwal R. The role of push-pull technology in privacy calculus: the case of location-based services. Journal of management information systems. 2009 Dec 1;26(3):135-74.
9. Bhattacherjee A. Understanding information systems continuance: An expectation-confirmation model. MIS quarterly. 2001 Sep 1:351-70.
10. Gaskin J, James M, Lim J, Steed J. Master Validity Tool, AMOS Plugin. 2023. Gaskination's StatWiki.
11. Straub D, Boudreau MC, Gefen D. Validation guidelines for IS positivist research. Communications of the Association for Information systems. 2004;13(1):24.
12. Kline E, Wilson C, Ereshefsky S, Tsuji T, Schiffman J, Pitts S, Reeves G. Convergent and discriminant validity of attenuated psychosis screening tools. Schizophrenia research. 2012 Jan 1;134(1):49-53.
13. Fornell C, Larcker DF. Evaluating structural equation models with unobservable variables and measurement error. Journal of marketing research. 1981 Feb;18(1):39-50.
14. Petter S, Straub D, Rai A. Specifying formative constructs in information systems research. MIS quarterly. 2007 Dec 1:623-56.
